# Ticagrelor Associated Heart Block: The Need for Close and Continued Monitoring

**DOI:** 10.1155/2017/5074891

**Published:** 2017-01-26

**Authors:** Munish Sharma, Daniel A. N. Mascarenhas

**Affiliations:** ^1^Internal Medicine, Easton Hospital, Easton, PA, USA; ^2^Drexel University College of Medicine, Philadelphia, PA, USA; ^3^Easton Hospital, PA, USA

## Abstract

Ticagrelor is an antiplatelet agent prescribed to prevent the development of adverse cardiac events after acute coronary syndrome (ACS). According to the PLATO trial, ticagrelor is associated with ventricular pauses in the first week of treatment; however, these episodes were felt to be asymptomatic and nonfatal to the patient. We present a case of ticagrelor related second-degree type II heart block causing severe dizziness and diaphoresis that resolved after discontinuation of the medication.

## 1. Introduction

It is mandatory to start a patient with ACS with or without ST-segment elevation on dual antiplatelet therapy with aspirin and a P2Y12 platelet receptor inhibitor [1]. Amongst the P2Y12 platelet receptor inhibitors, the use of clopidogrel is hampered by the slow and variable transformation of the prodrug to the active metabolite, modest and variable platelet inhibition, an increased risk of bleeding, and an increased risk of stent thrombosis [2]. Prasugrel, a thienopyridine prodrug, has good inhibitory effect on platelets and has lower risk of myocardial infarction and stent thrombosis, but it is associated with a higher risk of major bleeding in patients [2]. Ticagrelor, a reversible and direct-acting oral antagonist of the adenosine diphosphate receptor P2Y12, was found to provide faster and more efficacious P2Y12 inhibition than clopidogrel with no increased bleeding risk [3]. According to the PLATO trial, which established superiority of ticagrelor over clopidogrel in preventing major cardiovascular adverse events, there was increased incidence of ventricular pause and dyspnea with ticagrelor in the first week of treatment compared to those receiving clopidogrel (5.8% versus 3.6%, resp.; *p* = 0.01). But such episodes were concluded to be infrequent or the same as clopidogrel at 30 days (2.1% versus 1.7%, resp., for ticagrelor and clopidogrel; *p* = 0.52) and were rarely associated with symptoms [4]. We present a case of second-degree type II heart block due to ticagrelor which was diagnosed 6 months after initial percutaneous coronary intervention (PCI) of left circumflex artery done for unstable angina. This case report will also add to the existing literature about incidences of symptomatic atrioventricular block (AV block) that can occur with ticagrelor.

## 2. Case Description

**Figure 1:**
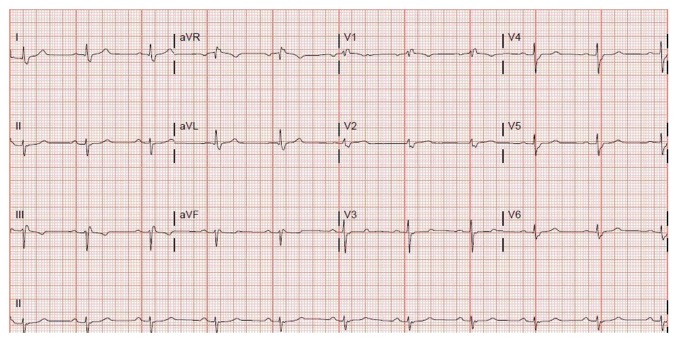
EKG showing borderline AV conduction delay and right bundle branch block (RBBB) pattern.

**Figure 2:**
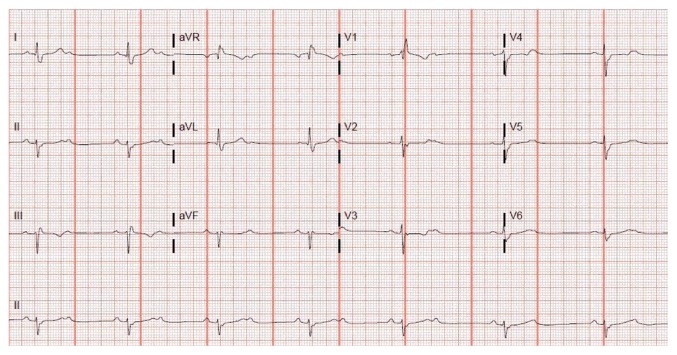
EKG showing second-degree type II heart block.

**Figure 3:**
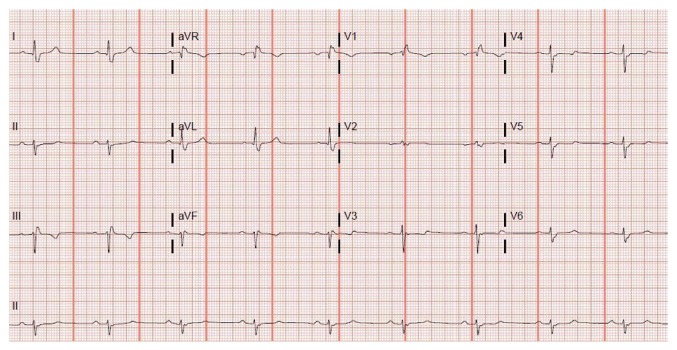
EKG showing sinus bradycardia with borderline AV conduction delay with RBBB pattern.

In May of 2016, a 55-year-old male with history of hypertension, hyperlipidemia, and coronary artery disease (CAD) status after angioplasty of left anterior descending (LAD) artery 2 years ago presented to the emergency room (ER) with substernal chest pain. His cardiac enzymes were not elevated and electrocardiogram (EKG) showed normal sinus rhythm at a rate of 62 bpm with borderline AV conduction delay and right bundle branch block (RBBB) pattern (Figure 1). The patient was only taking aspirin and had discontinued his statin due to muscle ache and metoprolol because of fatigue. His chest pain was relieved with sublingual nitroglycerin in the ER. In view of his significant history of CAD and typical anginal symptoms, patient underwent cardiac catheterization. It revealed 90 percent stenosis of the left mid circumflex coronary artery which was successfully stented with a drug-eluting stent. Right coronary artery (RCA) showed 50–60% stenosis while LAD showed patent stent. The patient recovered very well. After calculating the GRACE score, the patient was discharged on aspirin and ticagrelor as per the ACC guidelines. He was also prescribed rosuvastatin and metoprolol. Two months after PCI, the patient presented to his primary cardiologist due to worsening fatigue and intermittent dizziness. His metoprolol was stopped. In November, 2016, he was brought by emergency squad to the hospital due to worsening dizziness and diaphoresis at rest. He denied any h/o chest pain, palpitations, nausea, or loss of consciousness. The patient had stopped ticagrelor 1 day prior to this admission since he ran out of it. The patient requested a second opinion on this admission. On physical examination, his blood pressure was 126/66 mmHg and heart rate was regular at 42 beats per minute. The neck was supple with no carotid bruits and cardiac auscultation revealed 2/6 ejection systolic murmur in the left sternal border. Patient's EKG revealed second-degree type II atrioventricular (AV) block which was new (Figure 2). His cardiac enzymes, thyroid stimulating hormone, serum potassium, and magnesium were within normal range. The patient did not receive ticagrelor further during the admission. Cardiac catheterization revealed RCA stenosis of 60–70% but no intervention was required as this was not the cause of the new onset heart block. We considered starting patient on oral theophylline on admission had the heart block not resolved within 24 hours. His second-degree heart block resolved 2 days after discontinuation of ticagrelor and his EKG showed sinus bradycardia (heart rate = 54 bpm) with borderline AV conduction delay with RBBB pattern (Figure 3) which was similar to his baseline EKG. Clopidogrel was started as an alternative P2Y12 platelet receptor inhibitor and aspirin was continued. He was discharged after an event monitor was placed.

## 3. Discussion

According to the PLATO trial, ventricular pauses associated with ticagrelor are believed to be of no clinical significance. But recent noticeable surge in case reports about severe symptomatic bradycardia with high grade AV block necessitates need for a well-structured larger study to investigate this potentially life threatening adverse effect of ticagrelor. A case control study conducted in 140 cases and 560 controls identified during the period of April 2012 to March 2014 found no significant association between bradycardia and exposure to ticagrelor relative to clopidogrel in the previous 90 days prior to the index date [5]. But more data exuding from larger studies would be useful to strengthen such findings.

The mechanism of bradyarrhythmia due to ticagrelor is not well established. Ticagrelor inhibits cellular uptake of adenosine and thus increases its plasma concentration which can cause AV blocking effect. It is also thought to affect the cardiac automaticity and conduction [6, 7]. Prior case reports have emphasized the tendency of developing high grade AV blocks with ticagrelor in patients with preexisting conduction defect and in those on medications with AV nodal blocking properties. Ticagrelor is already under the Food and Drug Administration (FDA) scanner for a potential serious AV block as reported in the FAERS database, October 4th, 2016. Our patient also had a first-degree AV block at baseline and was on metoprolol which was discontinued in spite of which he developed Mobitz type II block 6 months after PCI which was completely new. His clinical and electrocardiogram findings improved only after discontinuation of ticagrelor.

There is contradictory view about the need to place permanent pacemaker in patients with high grade AV block due to ticagrelor. Some authors have reported that they needed to implant permanent dual chamber pacemaker in their patient 10 days after discontinuation of ticagrelor since the patient was symptomatic [8]. While others have reported that second-degree AV block returned to first degree on merely discontinuing ticagrelor. Routine use of temporary or permanent pacemaker is not indicated unless patient is symptomatic [9]. In our case, we discontinued the ticagrelor and patient showed significant improvement in his symptoms. Insertion of temporary or permanent pacemaker was not required.

## 4. Conclusion

Extreme caution and close monitoring for development of heart block are needed after initiation of ticagrelor, especially in patients with preexisting conduction defect or on AV nodal blocking agent. Beta blockers may not be the only reason for such cases of symptomatic bradycardia or high grade AV block. Ticagrelor should be considered as the possible offending agent. Other P2Y12 platelet receptor inhibitors such as clopidogrel or prasugrel are suitable alternatives if the patient is at risk for development of a potentially life threatening heart block.
